# Increased Expression of IL-22 Is Associated with Disease Activity in Behcet’s Disease

**DOI:** 10.1371/journal.pone.0059009

**Published:** 2013-03-19

**Authors:** Tao Cai, Qian Wang, Qingyun Zhou, Chaokui Wang, Shengping Hou, Jian Qi, Aize Kijlstra, Peizeng Yang

**Affiliations:** 1 The First Affiliated Hospital of Chongqing Medical University, Chongqing, P.R. China; 2 Chongqing Eye Institute, Chongqing, P.R. China; 3 Chongqing Key Laboratory of Ophthalmology, Chongqing, P.R. China; 4 University Eye Clinic Maastricht, Maastricht, The Netherlands; University Medical Center Freiburg, Germany

## Abstract

**Objective:**

Interleukin (IL)-22 has been reported to be involved in the development of autoimmune diseases. This study aimed to analyze the expression and potential role of IL-22 in the pathogenesis of Behcet’s disease (BD).

**Methods:**

The levels of IL-22 in patient sera or supernatants of cultured peripheral blood mononuclear cells (PBMCs) and CD4^+^T cells were detected by enzyme-linked immunosorbent assay (ELISA). Flow cytometry was used to evaluate the frequency of IL-22–producing CD4^+^ T cells. IL-22 mRNA from erythema nodosum skin lesions was examined using real time quantitative RT-PCR.

**Results:**

BD patients with active uveitis showed a significantly higher expression of IL-22 in the supernatants of stimulated PBMCs and CD4^+^T cells compared with BD patients without active uveitis and normal controls. An increased frequency of IL-22-producing CD4^+^T cells was also found in BD patients with active uveitis. IL-22 mRNA expression was elevated in erythema nodosum skin lesions. In BD patients, a high IL-22 level in the supernatant of stimulated PBMCs correlated with the presence of retinal vasculitis and erythema nodosum.

**Conclusions:**

IL-22 was associated with disease activity in BD and correlated with the presence of small vessel inflammation, suggesting that it may be involved in its pathogenesis.

## Introduction

Interleukin (IL)-22, a member of the IL-10 superfamily, is primarily produced by activated T cells and natural killer (NK) cells. [1]–[2] It is often secreted together with IL-17 by Th17 cells. [3]–[4] IL-22 mediates its effect through 2 receptors: IL-10Rb, which is ubiquitously expressed and the heterodimeric receptor IL-22R1, which is restricted to nonlymphoid cells such as epithelial cells and fibroblasts. [2], [5] Upon binding to its receptor, IL-22 can initiate inflammatory immune responses and has been shown to induce production of CCL-2 and proliferation of synovial fibroblasts and osteoclasts in rheumatoid arthritis (RA). [6]–[7] Recent studies have shown that IL-22 may be involved in the pathogenesis of autoimmune diseases including psoriasis, Crohn`s disease (CD), rheumatoid arthritis (RA), Sjögren’s syndrome and systemic lupus erythematosus (SLE). [8]–[12] Earlier studies showed that IL-22 mRNA was highly expressed by PBMCs from noninfectious uveitis patients [13].

IL-22 is also involved in the survival of cells in the liver, lungs and gut and may thus have a protective effect besides its proinflammatory activity.[14]–[16] It has for instance been shown to be protective against experimental hepatitis and Crohn`s disease (CD) [17].

Behcet’s disease (BD) is a chronic systemic inflammatory disease characterized by recurrent uveitis, oral aphthae, genital ulcers, and skin lesions. Although the pathogenesis of BD is still unclear, several reports have suggested that autoinflammatory mechanisms may play a crucial role. Recent studies from our group have suggested that Th17 plays an active role in this disease. [18] As yet, it is not clear whether IL-22, one of Th17 cells secreting cytokines, is also involved in this disease. The study presented here was designed to examine the expression of IL-22 in BD, and it`s association with clinical features. Our results showed that IL-22 was associated with the activity of BD and the occurrence of small vessel inflammation, suggesting it may be involved in its pathogenesis.

## Materials and Methods

### Patients

Thirty-eight BD patients (31 men, 7 women) referred to us from October 2009 to May 2011, with an average age of 33.7 years, and twenty-one healthy persons (16 men, 5 women), with an average age of 30.5 years, were included in this study (table 1). The diagnosis of BD was based on the diagnostic criteria designed by the International Study Group for BD. [19]–[20] Twenty patients showed active recurrent intraocular inflammation evidenced by dust keratic precipitates (100%), flare and cells in the anterior chamber (100%) and hypopyon (35%), and retinal vasculitis observed clinically or disclosed by fluorescein angiography (100%). The extraocular manifestations during the entire course of the disease included recurrent oral aphthous lesions (100%), multiform skin lesions (69%) or recurrent genital ulcers (40%), and arthritis (36%). None of these active patients received immunosuppressive drugs for at least one week before visiting us and blood drawing. Eighteen patients showed inactive intraocular inflammation after treatment with prednisone combined with cyclosporin A for more than 3 months. Fifteen inactive patients did not receive any immunosuppressant for at least 2 months while three inactive patients had used a small dose of oral prednisone (5 mg every other day) up to one month before blood sampling. A separate group of uveitis patients with acute anterior uveitis (AAU) (n = 12) were included as controls (table 1) to evaluate the expression of IL-22 in another active uveitis entity.

**Table 1:** Clinical characteristics of BD patients, AAU patients and normal controls.

Parameters	normal controls(n = 21)	active BD patients(n = 20)	inactive BD patients(n = 18)	AAU patients(n = 12)
age (years)	30.5±6.6	31.4±10.9	36.2±14.5	34.6±15.2
female/male (n)	16/5	16/4	15/3	5/7
cells in the anterior chamber	NA	20/20	0/18	12/12
flare in the anterior chamber	NA	20/20	0/18	12/12
hypopyon	NA	7/20	0/18	4/12
vitreous opacity	NA	20/20	9/18	0/12
retinal vasculitis	NA	20/20	#4/18	0/12
retinal edema	NA	20/20	0/18	0/12
*oral ulcer	NA	3/20	1/18	0/12
*genital organ ulcer	NA	1/20	1/18	0/12
*erythema nodosum	NA	4/20	1/18	0/12
*arthritis	NA	2/20	1/18	0/12
CRP (mg/dl)	ND	2.8±3.9	2.2±2.7	2.6±3.4
ESR (mm/h)	ND	32.9±27.3	16.5±7.4	27.6±21.2
&visual acuity (LogMAR)	ND	0.62±0.47	0.81±0.65	0.77±0.61
&intraocular pressure(mmHg)	ND	28.4±17.5	15.3±6.7	19.0±10.3

Data are shown as mean±SD or absolute numbers.

# No detectable retinal vasculitis clinically but presence of dye leakage from retinal blood vessels in 4 inactive BD patients was disclosed by fluorescein angiography.

* Extraocular lesions observed at the time of blood sampling are shown in this table. Patients whose extraocular lesions were negative may have experienced these symptoms during an earlier phase of the disease.

& Visual acuity was tested with Logarithm of mininal angle of resolution acuity chart, intraocular pressure was measured with Non-contact tonometer.

BD, Behcet disease, AAU, acute anterior uveitis.

NA, not applicable, ND, not determined.

CRP, C-reactive protein, ESR, erythrocyte sedimentation rate;

### Ethics Statement

Written informed consent was obtained from each participant, and this study was approved by the Clinical Research Ethics Committee of the First Affiliated Hospital of Chongqing Medical University (Permit Number: 2009–201008) and adhered to the tenets of the Declaration of Helsinki.

### Cell Isolation and Culture

Anticoagulated blood samples were obtained using vacuum tubes containing EDTA. Peripheral blood mononuclear cells (PBMCs) were prepared by Ficoll-Hypaque density-gradient centrifugation. CD4^+^T cells were purified by MACS using a human CD4^+^ T cell isolation kit (eBioscience, San Diego, CA). PBMCs were stimulated with anti-CD3 (5 ug/ml; eBioscience, San Diego,CA) and anti-CD28 antibodies (1 ug/ml; eBioscience) for 72 hours at a density of 2×10^6^cells/ml. CD4^+^T cells were stimulated with anti-CD3 (5 ug/ml; eBioscience) and anti-CD28 antibodies (1 ug/ml; eBioscience) for 72 hours at a density of 1×10^6^cells/ml.

### Measurement of Cytokines by ELISA

The concentration of IL-22 in sera and cell culture supernatants of patients and controls was assayed using a Duoset human IL-22 enzyme-linked immunosorbent assay (ELISA) kit (R&D Systems,Minneapolis,MN) with a detection limit of 15.6 pg/ml.

### Intracellular Cytokine Staining

A fluorescence-activated cell sorter (FACS) (Beckman Coulter, CA, USA) was used to analyze the cell surface phenotype (CD4, CD8) and intracellular IL-22 and IL-17 production by PBMCs. In total, 2×10^6^ cells/ml PBMCs were stimulated with 20 ng/ml phorbol 12-myristate 13-acetate (Sigma-Aldrich,St Louis Mo) and 1 ng/ml ionomycin (Sigma) for 5 hours. During the last hour, 10 ug/ml Brefeldin A (Sigma) was added to the cultured PBMCs. The stimulated PBMCs were washed in PBS and fixed in 4% formaldehyde, permeabilized with 0.1% saponin (Sigma), and stained with Percp-cy5.5-labeled anti-CD3 (BD Pharmingen, San Diego, CA), phycoerythrin (PE)-cy7–labeled anti-CD8, FITC-labeled anti–IL-22, PE-labeled IL-17 or a matched isotype control mAb (eBioscience). Flow cytometry was conducted on FACS Aria, and the data were analyzed with FACSDiva Software (BD Bioscience, San Diego, CA).

### Quantitative Real-time PCR

To determine the mRNA expression of IL-22 in skin lesions, quantitative real-time PCR was performed. First of all, biopsies were obtained from erythema nodosum skin lesions of BD patients and normal skin samples from healthy individuals (table 2). All skin samples were deep and went into the subcutaneous fat layer. The biopsies were snap-frozen in liquid nitrogen before being stored at −80°C for later RNA extraction. Each sample was lysed in a tissue homogenizer, and RNA was extracted using the RNAeasy Mini Kit (Qiagen) according to the manufacturer’s instructions. Total RNA was reverse transcribed to cDNA using the Superscript III Reverse Transcriptase system (Invitrogen). Real-time quantitative PCR was performed on the iCycler (Biorad) using the Quanti Tect SYBR Green PCR kit (Applied Biosystems). The IL-22 mRNA was normalized to the expression of β-actin mRNA. Genetool software was used to design the primers and the accession number of the sequence used to generate the primers is NM-020525.4. The forward primers for IL-22 were 5′TGGCAAAGAAGGGCTGTCAG 3′ and reverse primers were 5′GCGGTGACCCTGGCATAGT 3′. The ABI Prism Sequence Detection System software was used to determine the mRNA expression of IL-22 relative to β-actin in each sample. The expression of IL-22 was detected as described previously [2].

**Table 2:** Clinical and immunological characteristics of the patients selected for skin biopsy.

Parameters	Patient1	Patient2	Patient3	Patient4	Patient5
age (years)	32	27	30	35	29
female/male	F	F	M	F	F
cells in the anterior chamber	+	+	+	+	−
flare in the anterior chamber	+	+	+	+	−
hypopyon	+	−	−	−	−
vitreous opacity	+	+	+	+	+
retinal vasculitis	+	+	+	+	−
retinal edema	+	+	+	+	−
oral ulcer	+	−	−	−	−
genital organ ulcer	−	−	−	−	−
erythema nodosum	+	+	+	+	+
arthritis	−	+	−	−	−
CRP (mg/dl)	5.4	4.5	2.9	3.6	1.8
ESR (mm/h)	53.6	26	21	41.4	19
IL-22 serum level(pg/ml)	224	186	145	344	129
IL-22 expression by activated PBMCs (pg/ml)*	1806	1654	1782	1845	1528
IL-22-positive CD4^+^T cells in PBMCs (%)*	3.1	3.7	2.7	2.5	1.5

Data are shown as mean±SD or absolute numbers.

* The expression of IL-22 in the supernatants of stimulated PBMCs from every BD patient with erythema nodosum (n = 5) is higher than the normal range (792–981 pg/ml, 95% confidence interval for mean), the requency of IL-22-positive CD4+ T cells in PBMCs from BD patients with erythema nodosum (n = 4) is also higher than the normal range (0.86%–1.6%, 95% confidence interval for mean).

### Statistical Analysis

One-way ANOVA, Student t-test and Fisher`s exact test were applied using SPSS 13.0. Data were expressed as mean±SD. A level of p<0.05 was considered to be statistically significant.

## Results

### Increased Expression of IL-22 in the Supernatants of Stimulated PBMCs and CD4^+^ T Cells from BD Patients with Active Uveitis

**Figure 1:**
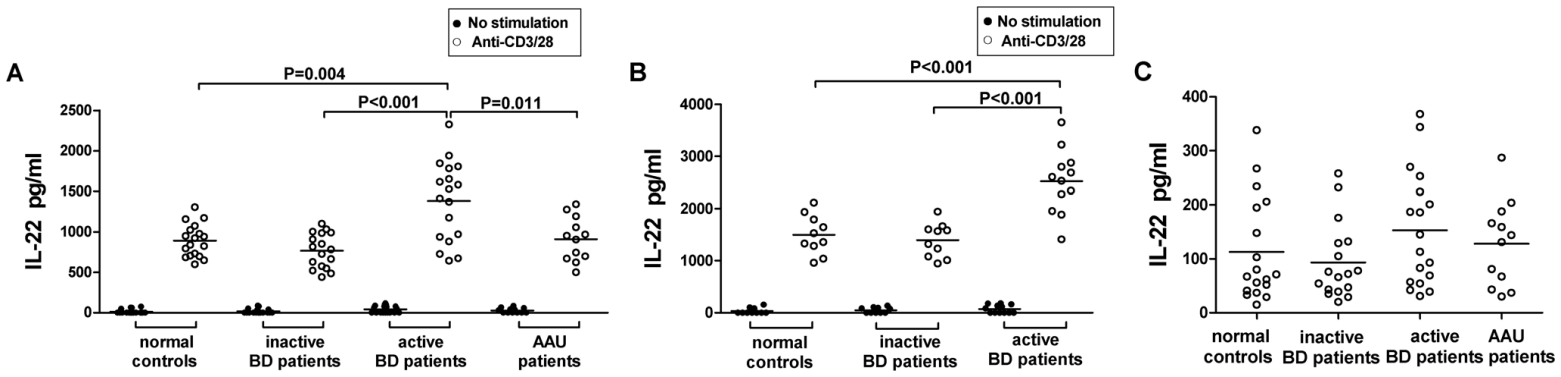
The expression of IL-22 in sera, supernatants of PBMCs and CD4^+^T cells of BD patients and normal controls. (A) IL-22 levels in the supernatants of cultured PBMCs after stimulation with or without anti-CD3 and anti-CD28 antibodies were significantly up-regulated in BD patients with active uveitis (n = 18) compared with that in BD patients without active uveitis (n = 17), AAU patients (n = 12) and normal controls (n = 18). (B) IL-22 secretion by purified CD4^+^T cells after stimulation with or without anti-CD3 and anti-CD28 antibodies was significantly up-regulated in BD patients with active uveitis (n = 12) compared with that in BD patients without active uveitis (n = 10) and normal controls (n = 10). (C) There were no significant differences with regards to IL-22 serum levels among BD patients with active uveitis (n = 18), BD patients without active uveitis (n = 17), patients with idiopathic acute anterior uveitis (AAU) (n = 12) and normal controls (n = 18). Data were expressed as mean±SD.

There was no significant difference with regards to IL-22 serum levels among BD patients with active uveitis (153±106 pg/ml, n = 18), BD patients without active uveitis (93±71 pg/ml, n = 17), AAU patients (128±79 pg/ml, n = 12) or normal controls (113±95 pg/ml, n = 18) (Fig. 1C). IL-22 protein levels in the supernatants of unstimulated PBMCs or unstimulated CD4^+^T cells were undetectable or only detected at a low level in BD patients and normal controls (Fig. 1A, 1B). After stimulation with anti-CD3 and anti-CD28 antibodies, a considerably higher concentration of IL-22 could be detected in PBMCs from BD patients with active uveitis (1382±489 pg/ml) compared to that observed in BD patients without active uveitis (778±211 pg/ml, p<0.001), AAU patients (911±271 pg/ml, p = 0.011) or normal controls (886±190 pg/ml, p = 0.004) (Fig. 1A). No significant difference was found between inactive patients, AAU patients and controls. Similar to the PBMCs, the production of IL-22 following stimulation of purified CD4^+^T cells was significantly higher in patients with BD disease with active uveitis (2520±607 pg/ml) than in patients with BD disease with inactive uveitis (1395±312 pg/ml, p<0.001) or normal controls (1488±358 pg/ml, p<0.001) (Fig. 1B).

### Increased IL-22-producing CD4^+^ T Cells in BD Patients with Active Uveitis

**Figure 2:**
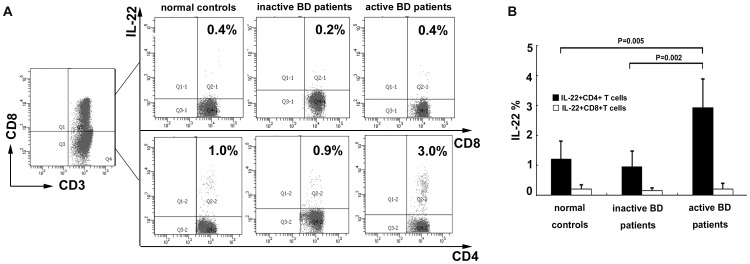
The frequency of IL-22-expressing T cells from BD patients with (n = 10) or without (n = 8) active uveitis and normal controls (n = 10). (A) Representative flow cytometry dot plots showing the expression of IL-22. Values indicate the cell frequencies in the respective quadrants. (B) Percentages of IL-22–producing CD4^+^ T cells were significantly higher in BD patients with active uveitis compared with that in BD patients without active uveitis and normal controls. There was no significant difference regarding the frequencies of IL-22-positive CD8^+^ T cells among BD patients with or without active uveitis and normal controls. Data were expressed as mean±SD.

Given the significantly increased IL-22 in the supernatants of stimulated CD4^+^ T cells, we further analyzed the frequency of IL-22-producing T cells in PBMCs obtained from BD patients and normal controls using FACS analysis. Both CD4^+^ T and CD8^+^ T cells scarcely expressed IL-22 without stimulation. CD4^+^T cells abundantly expressed IL-22 after stimulation with PMA and ionomycin. Furthermore, the frequency of IL-22 positive CD4^+^ T cells from BD patients with active uveitis (2.8%±1.14%) was significantly higher than from BD patients without active uveitis (0.97%±0.44%, p = 0.002) or normal controls (1.23%±0.51%, p = 0.005) (Fig. 2A, 2B). Our results also showed that the majority of IL-22 expressing T cells were CD4^+^T cells and only 10%–16% of the IL-22 positive T cells were CD8^+^ cells. Moreover, there was no significant difference regarding the frequency of IL-22-positive CD8^+^ T cells among BD patients with or without active uveitis and healthy controls (Fig. 2A, 2B).

**Figure 3:**
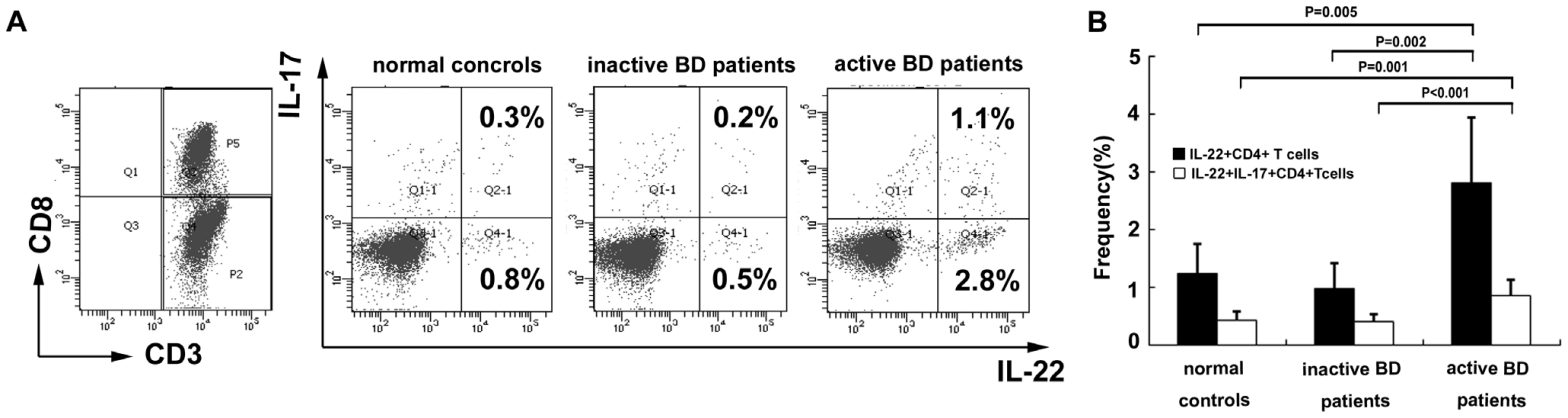
The percentages of IL-22/IL-17 double positive CD4^+^T cells from BD patients with (n = 8) or without (n = 8) active uveitis and normal controls (n = 8). (A) Representative flow cytometry dot plots for expression of IL-22 and IL-17. Values indicate the cell frequencies in the respective quadrants. (B) The frequency of IL-22/IL-17 double positive CD4^+^T cells from BD patients with active uveitis was significantly higher than from BD patients without active uveitis and normal controls. Only 31%–41% of IL-22-positive CD4+T cells were also positive for IL-17 in both BD patients and normal controls. Data were expressed as mean±SD.

To investigate the co-expression of IL-22 and IL-17 we performed FACS analysis on a purified population of CD4^+^T cells. The results showed that the frequency of IL-22/IL-17 double positive CD4^+^T cells from BD patients with active uveitis (0.86%±0.27%) was significantly higher than in BD patients without active uveitis (0.40%±0.13%, p<0.001) or normal controls (0.43%±0.15%, p = 0.001) (Fig. 3A, 3B). However, only a part of the IL-22-positive CD4^+^T cells was also positive for IL-17 in BD patients with active uveitis or without active uveitis and normal controls (31%–41%). More than half of the IL-22-producing cells were negative for IL-17 (Fig. 3A, 3B).

### Increased Expression of IL-22 mRNA in Erythema Nodosum Skin of BD Patients

**Figure 4:**
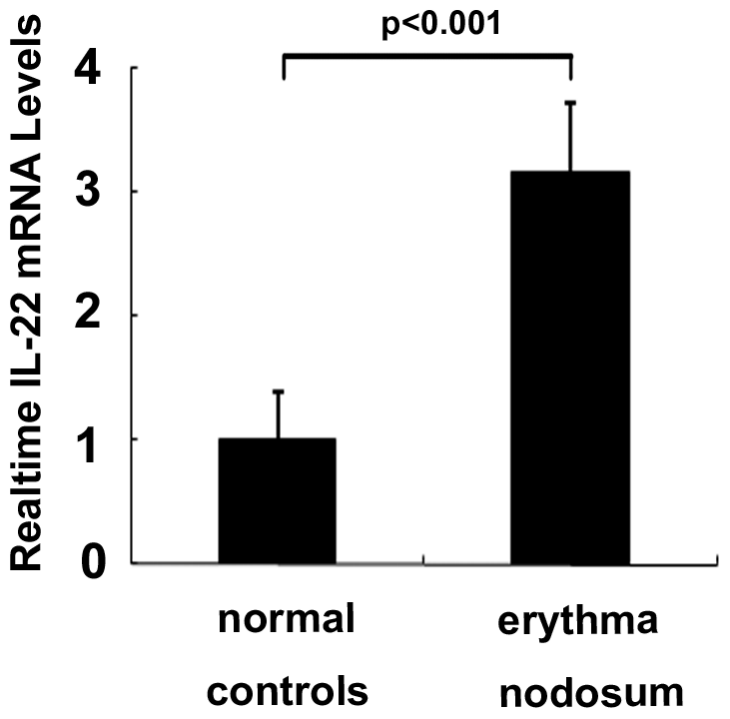
Expression of IL-22 mRNA in the erythema nodosum skin lesion from BD patients (n = 5) and normal control skin (n = 5). Data were expressed as mean±SD.

Expression of IL-22 in skin lesions, was investigated by examining IL-22 mRNA in erythema nodosum lesions obtained from BD patients. Results showed that the expression of IL-22 mRNA in erythema nodosum skin was significantly higher in BD patients than in normal skin (p<0.001) (Fig. 4). Consistent with the increased expression in skin lesions, the expression of IL-22 in the supernatants of stimulated PBMCs and the frequency of IL-22-positive CD4+ T cells in PBMCs from BD patients with erythema nodosum were also higher than the normal range (table 2).

### Correlations between Clinical Features and the IL-22 Levels in the Supernatants of Stimulated PBMCs from BD Patients

The results presented above showed that expression of IL-22 correlated with disease activity in BD. To further investigate the correlation between clinical, serological parameters and the expression of IL-22 in BD patients, two groups of patients were formed within the BD cohort. A first group with IL-22 levels within the normal range was denoted as ‘normal IL-22’ (792–981 pg/ml, 95% confidence interval for mean in normal controls), and a second group with markedly elevated IL-22 levels in the supernatants of stimulated PBMCs was designated as ‘high IL-22’ (>981 pg/ml) (table 3).

**Table 3:** Groups according to IL-22 expression by activated PBMCs from patients with BD.

Groups	active BD (n)	inactive BD (n)
**IL-22 normal** (792–981 pg/ml) (n = 18)	6	12
**IL-22 high** (>981 pg/ml) (n = 17)	12	5

The results showed that parameters such as “proportion of cells in the anterior chamber”, retinal vasculitis and erythema nodosum were significantly greater in the ‘high IL-22’ group than in the ‘normal IL-22’ group (p = 0.044, p = 0.005, p = 0.019 respectively). However, the two groups did not differ significantly for other clinical data, such as hypopyon, vitreous opacity, oral ulcer, genital ulcer, CRP levels and ESR levels (table 4).

**Table 4:** Clinical and serological parameters in BD patients in the ‘IL-22 normal’ group and ‘IL-22 high’ group.

Parameters	‘IL-22 normal’ (n = 18)	‘IL-22 high’ (n = 17)	p Value
IL-22 (pg/ml)	710±161	1479±393	p<0.001
Cells in the anterior chamber (%)	33.3	70.6	p = 0.044
Hypopyon (%)	16.6	17.6	NS
Vitreous opacity (%)	72.2	82.3	NS
Retinal vasculitis (%)	38.8	88.2	p = 0.005
Oral ulcer (%)	11.1	11.7	NS
Genital organ ulcer (%)	5.6	5.8	NS
Erythema nodosum (%)	0	29.4	p = 0.019
CRP (mg/dl)	2.3±3.0	2.6±3.8	NS
ESR (mm/h)	28.6±25.4	33.5±30.6	NS
visual acuity	0.83±0.37	0.77±0.49	NS
intraocular pressure	19.4±11.5	26.3±15.8	NS

Data are shown as mean±SD or absolute numbers.

p values were calculated between the ‘IL-22 normal’ and ‘IL-22 high’ group by Fisher’s exact test.

NS, not significant.

## Discussion

The present study showed that IL-22 production by activated PBMCs and CD4^+^T cells was markedly increased in BD patients with active uveitis as compared to controls. IL-22 mRNA in erythema nodosum skin lesions obtained from BD patients was significantly higher than normal control skin. These results indicate that an enhanced expression of IL-22 correlates with disease activity in BD. An earlier gene expression profiling study by the group of Nussenblatt showed that IL-22 was the gene that showed the highest differential upregulated mRNA expression in noninfectious uveitis patients. [13] Our study extended these findings in a specific uveitis entity (BD) and focused on IL-22 protein expression by PBMCs and isolated CD4+ cells. We did not study IL-22 mRNA expression by PBMCs and in contrast to the Nussenblatt study we had to stimulate the T cells to generate IL-22 protein expression. In the Nussenblatt study five of the six investigated donors did not have a detectable IL-22 transcript and 1 donor showed a very low IL-22 mRNA expression. Of the 40 uveitis patients tested in this study, 15 had no detectable IL-22 mRNA expression, which is consistent with our data showing undetectable IL-22 protein expression by unstimulated PBMCs. The use of stimulated cells may provide a better reflection of the microenvironment at sites of inflammation.

IL-22, a member of the IL-10 cytokine superfamily, has been implicated in the pathogenesis of several autoimmune or autoinflammatory diseases. [8]–[13] To test whether IL-22 is involved in the development of BD, we evaluated the expression of IL-22 in BD patients, AAU patients and normal controls. We first tested serum levels of IL-22. No difference was observed between the various groups of patients and controls tested. Next we assayed IL-22 production by PBMCs. Significantly higher IL-22 expression was seen in supernatants of activated PBMCs from BD patients with active uveitis compared with BD patients without active uveitis, AAU patients and normal controls. The increased IL-22 expression observed in our study seemed to be unique to active BD in view of its normal expression in AAU patients although its expression in other uveitis entities still needs to be investigated.

Because CD4^+^ T cells play an essential role in the development of autoimmune diseases, we further measured the production of IL-22 by purified CD4^+^ T cells. The results showed a significantly elevated IL-22 production by activated CD4^+^ T cells in BD patients with active uveitis. Of interest was the observation that the amount of IL-22 produced by CD4^+^ T cells was 1.68–1.82 folds higher as compared to the same numbers of PBMCs irrespective whether cells were obtained from BD patients or normal controls. The findings indicated that CD4^+^T cells are the main cells responsible for IL-22 production in PBMCs. This conclusion was supported by FACS analysis, that showed that the majority of IL-22 was produced by activated CD4^+^T cells in PBMCs and that the contribution of CD8^+^T cells was only small. These experiments also showed that the frequency of IL-22 -positive CD4^+^ T cells was significantly higher in BD patients with active uveitis. The results are consistent with those reported earlier, showing that IL-22^+^CD4^+^T cells but not IL-22^+^CD8^+^T cells were significantly increased in patients with RA and SLE, and that their frequency correlated with disease activity. [12], [21] Although earlier reports suggested that IL-22 was co-expressed with IL-17, our data showed that only 31%–41% of the IL-22 positive CD4^+^T cells also expressed IL-17. These results revealed that, besides Th17, a large proportion of IL-22 is produced by other Th subtypes. These other subtypes may also be involved in BD pathogenesis, which is similar to observations found in patients with psoriasis [8].

Our finding showing an enhanced expression of IL-22 in BD is in line with reports showing an increased IL-22 expression in serum, PBMCs or CD4^+^T cells in patients with psoriasis, RA, Crohn’s disease and primary Sjögren syndrome.[8]–[9], [12]–[13], [21]–[22] IL-22 has also been reported to be over-expressed in affected tissues of patients with these diseases, [6], [9]–[10], [23] which is similar to our finding showing an increased IL-22 mRNA expression in erythema nodosum skin lesions in BD patients. These data collectively support a role for IL-22 as a disease activity marker for several autoimmune diseases. Interestingly, Ye et al reported that serum IL-22 was significantly decreased in SLE patients while the frequencies of IL-22-producing CD4^+^ T cells in peripheral blood were significantly increased as compared to healthy controls. [12], [24] The authors suggested that this phenomenon might be due to the fact that dexamethasone (DEX) inhibited the production of IL-22 in these patients. We also found inconsistencies in serum IL-22 level differences between the groups investigated and the in vitro IL-22 response by PBMCs. However, our patients didn`t receive immunosuppressive drugs. It is also possible that differences in the catabolism of IL-22 in vivo between patients and controls leads to similar blood levels despite higher production rates in the patient group. Further studies are needed to address this issue.

We showed that high IL-22 expression by activated PBMCs from BD patients correlated with the presence of retinal vasculitis, and erythema nodosum which similarly had noted lymphocytic vasculitis change in histopathology. [25] Increased expression of IL-22 mRNA was also found in skin samples with erythema nodosum of BD patients. Further studies are needed to show whether a correlation exists between known inflammatory Th17 cytokines such as IL-6, IL-17, IL-21, and IL-22 expression, both in skin as well as in PBMCs.

A recent study reported that IL-22 could affect human brain-derived microvascular endothelial cell permeability, and that it could promote CD4^+^ lymphocytes transmigrating across the blood-brain barrier. [26] These results suggest a possible involvement of IL-22 in the pathophysiology of vessel inflammation. IL-22 has also been shown to target epithelial cells and in vitro studies have shown that it can affect the physiological integrity of the primary fetal retinal epithelium cell cultures and can induce apoptosis of these cells. [13] We were not able to reproduce these findings using the ARPE-19 cell line, but this may be due to differences between the source of RPE cells used [27].

There is also some controversy concerning the role of IL-22 in uveitis. As mentioned above the group of Nussenblatt from the NIH have clearly shown an upregulated expression of IL-22 in clinical uveitis. [13] A recent study in mice undergoing experimental autoimmune uveitis however showed that IL-22 administration was able to suppress uveitis in these animals. [28] The discrepancy with the clinical findings is not clear and deserves further study.

In conclusion, our study showed an elevated expression of IL-22 by stimulated PBMCs and CD4^+^T cells, increased frequencies of IL-22-producing CD4^+^T cells in BD patients with active uveitis, and an increased expression of IL-22 mRNA in erythema nodosum skin lesions of BD patients and point to a possible role of this lymphokine in the pathogenesis of this disease.
